# Waiver of informed consent in clinical research: a summary of contemporary guidelines and a resource for researchers

**DOI:** 10.1136/bmjopen-2024-091896

**Published:** 2025-03-18

**Authors:** Amanda Siriwardana, Brendan Smyth, Meg Jardine, On behalf of the RESOLVE Study Global Team, Grace Balicki

**Affiliations:** 1 Sydney Medical School, Faculty of Medicine & Health, University of Sydney, Sydney, New South Wales, Australia; 2 The George Institute for Global Health, University of New South Wales, Sydney, New South Wales, Australia; 3 Department of Renal Medicine, Royal North Shore Hospital, Sydney, New South Wales, Australia; 4 NHMRC Clinical Trials Centre, Faculty of Medicine & Health, University of Sydney, Sydney, New South Wales, Australia; 5 Department of Renal Medicine, St George Hospital, Sydney, New South Wales, Australia; 6 Department of Renal Medicine, Concord Repatriation General Hospital, Sydney, New South Wales, Australia

**Keywords:** STATISTICS & RESEARCH METHODS, MEDICAL ETHICS, Research Design, Clinical Trial

## Abstract

**Abstract:**

**Objective:**

Low-risk pragmatic clinical research such as learning health system research, quality assurance activities and comparative effectiveness studies are approaches to embedding clinical research within routine practice to generate generalisable evidence. Individual written informed consent may present a barrier to the feasibility and inclusiveness of such low-risk clinical research. Within an overarching moral and ethical framework, the form and requirements of consent vary in both clinical practice and research settings according to the context and level of risk. Within some low-risk research settings, waiver of consent may be appropriate.

**Analysis:**

We sought to describe contemporary national and international English-language guidelines pertaining to the use and oversight of waiver of consent in clinical research. We identify 14 guidelines including 1 international, 1 regional and 12 national statements, and summarise the principles in each for circumstances in which a waiver of consent is appropriate.

**Conclusion:**

While complete international harmonisation of policy may be neither realistic nor necessary, there are numerous unifying concordances suggesting a broad consensus on the approach to waiver of consent research.

## Introduction

 Informed consent for research participation is a cornerstone of modern medical ethics and is an essential feature of randomised research involving novel therapies and technologies. While such studies spearhead progress in patient care, many aspects of the healthcare system are unsuited to individual randomised controlled trial designs or seek to assess the impact of differing treatment strategies or systems on broad patient populations that are often not fully captured by studies using traditional informed consent models. This is most relevant for pragmatic low-risk clinical research which aims to evaluate the effectiveness of interventions in real-world settings, often comparing the relative merits of medical treatments already in common use. Such research is often cited as key to achieving the goal of a ‘learning health system’, in which research is embedded within routine practice and the effectiveness of an intervention, practice or system change is assessed in all participants exposed.^1^ Comparative effectiveness pragmatic randomised controlled trials (RCTs) are a form of learning health system research, which act within areas of existing variation in clinical practice that are not justified by high-quality evidence or local conditions.^2 3^ These studies replace arbitrary treatment selection with randomised selection and are essential to improving patient outcomes, especially with respect to institutional or health system practices that are determined outside of, or prior to, individual clinician-patient treatment decisions.

Explanatory randomised trial populations have historically differed substantially from the broader patient population.^4^,^7^ These differences stem in part from inclusion and exclusion criteria that are designed both to protect participant safety and to define a population most suited to testing the study hypothesis. However, they also result from the informed consent process itself, which requires participants to understand consent material written in technical and legal style (or have a guardian able to do so), and to be sufficiently engaged in their healthcare so as to volunteer or accept referral for inclusion in research. These factors result in study populations which differ in important ways from the broader patient population, for example, being younger, less comorbid or with a better prognosis. Contrastingly, comparative effectiveness studies and evaluations of health-service interventions are designed for broader real-world settings and will naturally lead to changes in practice that affect all patients. Such studies will be most informative if they include generalisable populations, are sufficiently large and minimise bias. Given the barriers that traditional written informed consent models pose to inclusion of a representative patient population, such studies of practices where patient consent is not sought in *clinical practice* may warrant a similar waiver of *research* consent to minimise selection and other biases.^8^,^10^ The benefits of aligning consent requirements for practice evaluation with those for the conduct of clinical practice have long been recognised. In 1987, Chalmers *et al* posited that in the face of uncertainty in clinical research and clinical care, the consent processes required for rigorously conducted real-world studies compared with the lack of such requirements for clinical decision-making were an ethical discordance that impeded practice improvement.^11 12^ Recommendations for facilitating research and clinical practice integration are through the anchoring of consent requirements in a real-world study with those of the equivalent routine clinical practice setting.^13^,^16^

The increasing recognition of the impact of health services on clinical outcomes comes at a time when digital advancements are facilitating research and quality assurance studies that were previously technologically challenging or impossible.^2^ In this context, there is increasing interest in the utilisation of waiver of consent in low-risk clinical research. While the well-established opt-in consent model has broadly accepted advantages for participant autonomy and safety, researchers, ethics committees and the broader health community may benefit from a distillation of global guidance on situations in which it may be ethically acceptable to consider a waiver of consent. We therefore aimed to provide an overview of national and international statements of guidance on principles that outline when a waiver of consent model could be considered for an individual study.

## Methods

This analysis examines current international, regional and national English-language guidelines on ‘waiver of consent’ in clinical research within non-emergency settings. ‘Waiver of consent’ was defined as situations where investigators are not required to inform or request permission from research subjects prior to participation.^17^ Emergency care research was excluded as such research involves critically unwell individuals who are unable to consent and/or involve time-critical emergency interventions, for which modified or delayed consent is typically employed (ie, a temporary waiver of consent followed by seeking consent at a later date once the patient is stable, or from an authorised representative or guardian). The ethical principles relevant to such situations are distinct from that of waiver of consent in non-emergency clinical research.

We included all available English-language ethical guidelines or statements on the use of waiver of consent for non-emergency clinical research. We searched bibliographic databases MEDLINE and EMBASE using the search terms: ‘research’ or ‘clinical research’ or ‘medical research’ or ‘health research’, and ‘consent’ or ‘informed consent’ or ‘ethics’ or ‘medical ethics’, and ‘waiver’ or ‘simplified’. We also conducted an extensive online search for ‘grey literature’ using the above terms on the rationale that documents relevant to this review may be contained within health research regulatory guidelines produced by government agencies and not published in peer-reviewed literature.

### Patient and public involvement

Patients or the public were not involved in the design, conduct or reporting of this review.

## International, regional and national guidelines on waiver of consent

Our search yielded 14 eligible reference documents. None were obtained through database searches of MEDLINE and EMBASE, and all were obtained from ‘grey literature’ sources. These statements included 1 international and 13 national/regional guidelines.

**Figure 1:**
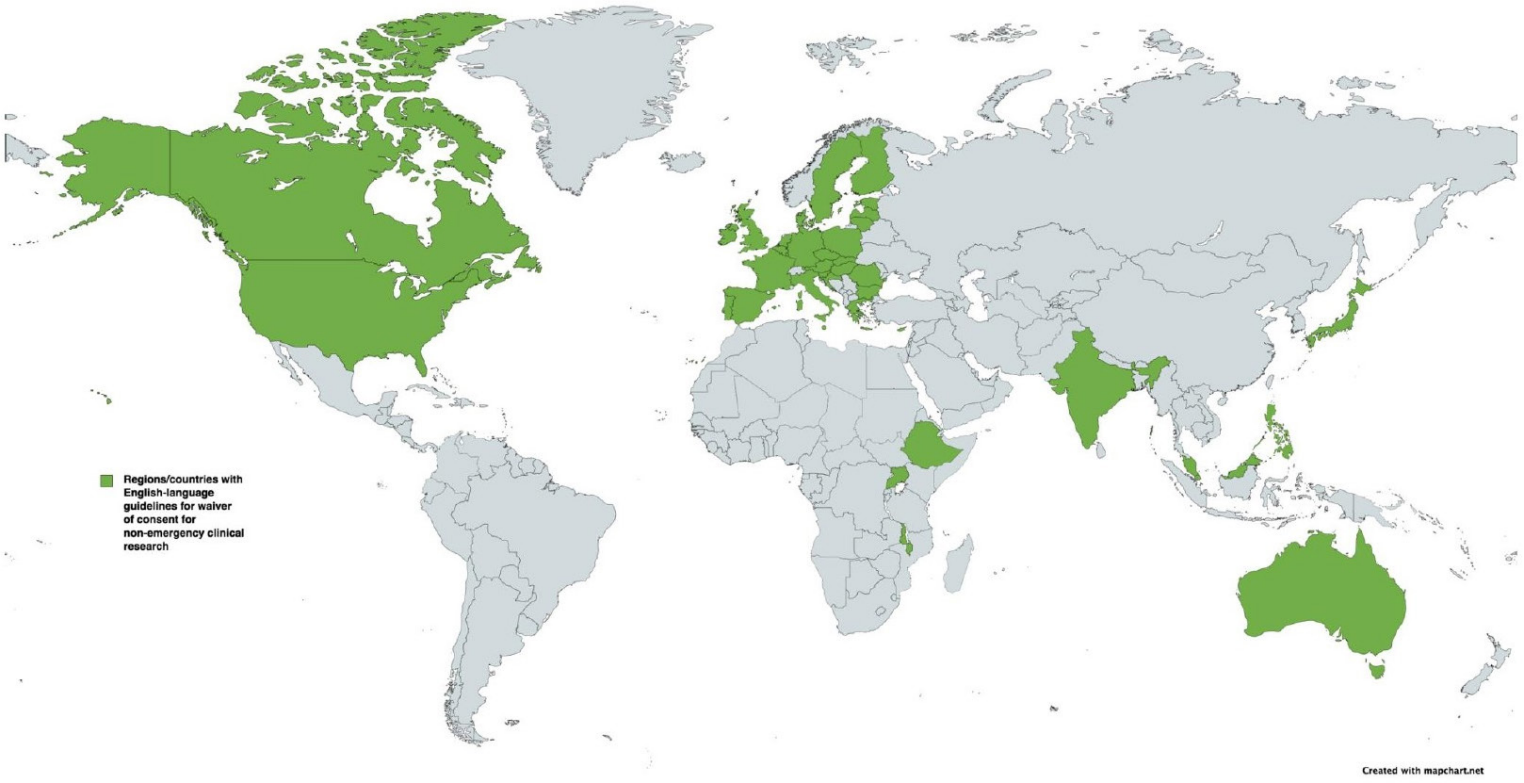
Countries with regional or national guidelines for ‘waiver of consent’ in non-emergency clinical research. Figure sourced from MapChart and modified with permission.

The *International Ethical Guidelines for Health-related Research Involving Humans* were first released in 1982 and have undergone several revisions, most recently in 2016.^18^ These international guidelines are a collaboration between the Council for International Organisations of Medical Sciences (CIOMS) and the WHO, and provide a global framework of ethical principles to guide the conduct of all health-related research involving human participants. Guideline 10 pertains to waiver of consent. In addition to the CIOMS international guideline, we identified 1 regional (European Union) and 12 national guidelines applicable in 39 countries (figure 1). These documents and their intended scope are detailed in table 1.

**Table 1:** International, regional and national guidelines included for review within this paper with ‘waiver of consent’ guidelines for non-emergency clinical research

Governing organisation(s) (region/country)	Guideline	Year of publication	‘Waiver of consent’ subsection	Scope of waiver of consent guideline
Council for International Organisations of Medical Sciences, CIOMS(International)	*International Ethical Guidelines for Health-related Research Involving Humans* ^18^	2016	Guideline 10	Applies to all clinical research, including ‘health-related research’, ‘studies [which] involve identifiable data or biological specimens’, ‘when data or biological specimens are not personally identifiable’, ‘studies [which] analyse existing data from health-related registries … for example, cancer registries and databanks of genetic or other anomalies’, and ‘when the participants are children, adolescents, and individuals not capable of giving informed consent’
US Department of Health & Human Services(USA)	*Code of Federal Regulations (CFR*)^17^	2019	Title 45: Public Welfare Part 46: Protection of Human Subjects (45 CFR 46)(Human research policy was initially defined in 1991 in 45 CFR 46 (‘The Common Rule’). The *IRB Waiver or Alteration of Informed Consent for Clinical Investigations Involving No More Than Minimal Risk to Human Subjects* was released in 2017 as an additional guideline for immediate implementation.^38^ This 2017 guideline was then included as a statutory change in the CFR, referred to as the ‘revised Common Rule’ and effective as of 21 January 2019^17^	Applies to all ‘clinical investigations’
Canadian Institutes of Health Research, Natural Sciences and Engineering Research Council of Canada, and Social Sciences and Humanities Research Council of Canada(Canada)	*Tri-Council Policy Statement: Ethical Conduct for Research Involving Humans* ^19^	2018	Articles 3.7A and 3.7B	Applies to all clinical research, with particular reference to ‘social sciences research’, use of ‘data and/or human biological materials’, ‘participants in vulnerable circumstances’, and ‘population and public health research’
European Parliament and Council(European Union)	*Regulation No 536/2014 On Clinical Trials on Medicinal Products for Human Use* ^30^	2014	Paragraph 33 and Article 30	Specifically for cluster randomised trials, where ‘the clinical trial requires that groups of subjects rather than individual subjects are allocated to receive different investigational medicinal products’
Health Research Authority, HRA (UK)	*Seeking informed consent for simple and efficient trials in the NHS (Draft guidance: For comment*)^20^	2014	Section 2.6(Although this remains a draft document, when put up for public and professional commentary, the majority of the 103 respondents supported the principles detailed in section 2.6^43^)	Applies to all clinical trials, with particular reference to pragmatic trials and cluster randomised trials
National Health and Medical Research Council, NHMRC(Australia)	*National Statement on Ethical Conduct in Human Research* ^21^	2018	Chapter 2.3.10	Applies to all clinical research using ‘personal information’ or ‘personal health information’
Bioethics Advisory Committee, BAC(Singapore)	*Ethics Guidelines for Human Biomedical Research* ^22^	2015	Paragraph 3.29	Applies to all ‘research done in the public interest’, with particular reference to ‘epidemiological or public health research carried out with medical records or with data from national registries’
Medical Review & Ethics Committee, MREC(Malaysia)	*Guidelines for Ethical Review of Clinical Research or Research Involving Human Subjects* ^23^	2006	Guideline 2	Applies to all clinical research
Philippine Health Research Ethics Board, PHREB(Philippines)	*National Ethical Guidelines for Health and Health-related Research* ^24^	2017	Paragraphs 15–17	Applies to all clinical research
Ministry of Health, Labour and Welfare, MHLW(Japan)	*Ethical Guidelines for Medical and Health Research Involving Human Subjects (English Edition*)^25^	2017	Chapter 5 Part 12 Paragraph 7	Applies to all clinical research
Indian Council of Medical Research, ICMR(India)	*National Ethical Guidelines for Biomedical and Health Research Involving Human Participants* ^26^	2017	Definitions of social value and minimal risk (sections 1 and 2) and criteria for waiver of consent research (section 5.7)	Numerous research examples are listed, including ‘retrospective studies, where the participants are deidentified or cannot be contacted’, ‘research on anonymised biological samples’, ‘public health studies/surveillance programmes/programme evaluation studies’, and ‘research on data available in the public domain’
Ministry of Science and Technology, MoST(Ethiopia)	*National Research Ethics Review Guideline (5th Edition*)^27^	2014	Article 6.15	Applies to all clinical research
National Health Sciences Research Committee, NHSRC(Malawi)	*General Guidelines on Health Research* ^28^	2007	Article 7.4	Applies to all clinical research
National Council for Science and Technology, UNCST(Uganda)	*National Guidelines for Research Involving Humans as Research Participants* ^29^	2014	Section 5.5	Applies to all clinical research

Within the CIOMS international guidelines, three essential criteria for using waiver of consent are described:

1. The research would not otherwise be feasible or practicable.
2. The research has important social value.
3. The research poses no more than minimal risk.

In addition to these criteria within the CIOMS guideline, other criteria were identified within national and regional guidelines for the use of waiver of consent and are summarised in table 2. Among these international, regional and national guidelines, we identified seven recurring principles which are discussed below.

**Table 2:** Individual criteria for waiver of consent in non-emergency clinical research within international, regional and national guidelines

Criterion	CIOMS (International)	CFR (USA)	Tri-Council (Canada)	European Parliament & Council (EU)	HRA (UK)	NHMRC (Australia)	BAC (Singapore)	MREC (Malaysia)	PHREB (Philippines)	MHLW (Japan)	ICMR (India)	MoST (Ethiopia)	NHSRC (Malawi)	UNCST (Uganda)
Research not otherwise feasible/practicable	+	+	+	+	+	+	+	+	+	+	+	+	+	+
Research has important social value	+	Δ	+	Δ		Δ	+		Δ	+	Δ	Δ		Δ
Research poses no more than minimal risk	+	+	+	+	+	+	+	+	+	+	+	+	+	+
Benefits from research justify any risks of harm associated with not seeking informed consent			+	+		+								
Intervention used in accordance with marketing and/or published scientific evidence				+	+									
Comparison between standard treatments				+	+									
Healthcare professionals can override the assigned intervention					+									
Protection of rights and welfare of subjects		+	+		+		+		+	+	+		+	
Participant has not expressed a preference for any particular treatment					+									
No reason to think participants would not have consented if asked						+								
Protection of privacy and confidentiality of subjects						+	+							
Provision of information after participation, where practicable, including potential to withdraw use of data where practicable		+	+	+		+			+	+		+	+	+
Involvement of community representatives prior to commencement, where practicable			+							+		+		
Subjects not deprived of financial benefits with commercial use of data						+								
Waiver is not prohibited by state, federal or international law				+		+								

Boxes in grey indicate criteria deemed essential in the 2016 CIOMS International Ethical Guidelines for Health-related Research Involving Humans guidelines. Use of ‘+’ denotes inclusion of a criterion within a country or region’s specific waiver of consent policy. Use of ‘Δ’ denotes inclusion of the ‘research of important social value’ criterion within a country or region’s broad clinical research policy though not specifically relating to waiver of consent research.

BACBioethics Advisory CommitteeCFRCode of Federal RegulationsCIOMSCouncil for International Organisations of Medical SciencesHRAHealth Research AuthorityICMRIndian Council of Medical ResearchMHLWMinistry of Health, Labour and WelfareMoSTMinistry of Science and TechnologyMRECMedical Review & Ethics CommitteeNHMRCNational Health and Medical Research CouncilNHSRCNational Health Sciences Research CommitteePHREBPhilippine Health Research Ethics BoardUNCSTNational Council for Science and Technology

## Review of individual principles

### Would not otherwise be feasible or practicable

Research being ‘not otherwise feasible or practicable’ was a universal criterion within all guidelines examined.^17^,^30^ ‘Unfeasible’ and ‘impracticable’ are specifically defined in some,^18 19^ with a consensus towards a lack of scientific or logistical feasibility and onerousness that wholly jeopardises study conduct; mere inconvenience is not a sufficient justification.^18 31 32^

This criterion is the central theme underpinning waiver of consent research, with particular emphasis that informed consent should remain the standard where it is practicable or feasible to obtain consent, regardless of whether other criteria are met for waiver of consent.^33 34^ Specific descriptions for this criterion within the examined guidelines are outlined in table 3.

**Table 3:** Detailed description of wording used for individual criteria contained within each international, regional and national guideline

1. Would not otherwise be feasible or practicable	
CIOMS (International)	‘the research would not be feasible or practicable to carry out without the waiver or modification’^18^
CFR (USA)	‘research could not practicably be carried out without the requested waiver’^17^
Tri-Council (Canada)	‘it is impossible or impracticable to carry out the research question properly, given the research design, if the prior consent of participants is required’^19^
HRA (UK)	‘following the normal consent process would place a disproportionate burden in terms of time and resources’^20^
NHMRC (Australia)	‘impracticable to obtain consent’^21^
BAC (Singapore)	‘the research could not practicably proceed without the waiver’^22^
MREC (Malaysia)	‘individual informed consent would make the conduct of the research impracticable’^23^
PHREB (Philippines)	‘the research cannot be practicably carried out without the waiver or alteration’^24^
MHLW (Japan)	‘if [informed consent procedures] are not omitted, it will be difficult to implement the research or the value of the said research will be significantly undermined’^25^
ICMR (India)	‘research cannot practically be carried out without the waiver and the waiver is scientifically justified’^26^
MoST (Ethiopia)	‘the research project could not practically be carried out without the waiver or alteration’^27^
NHSRC (Malawi)	‘the research could not practically be carried out without the consent waiver and obtaining informed consent is not practicable’^28^
UNCST (Uganda)	‘the research project could not practicably be carried out without the waiver or alteration’^29^
European Parliament & Council (EU) (in reference to cluster randomised trials only)	‘it is appropriate to allow that informed consent be obtained by simplified means for certain clinical trials where the methodology of the clinical trial requires that groups of subjects rather than individual subjects are allocated to receive different investigational medicinal products’^30^

2. Important social value and ‘risk-value’ assessment	
**2a. Social value**	
CIOMS (International)	‘the research has important social value’^18^
Tri-Council (Canada)	‘the potential benefits to be considered include benefits to the participants themselves, to the group they represent and/or to society more generally’^19^
BAC (Singapore)	‘research done in the public interest’^22^
MHLW (Japan)	‘the research to be implemented is recognised as being of socially high significance’^25^
CFR (US)(in reference to *all* clinical research)	‘research [is]… designed to develop or contribute to generalisable knowledge’^17^
European Parliament & Council (EU)(in reference to *all* clinical research)	‘low-intervention clinical trials are often of crucial importance … thereby optimising use of medicinal products and thus contributing to a high level of public health’^30^
NHMRC (Australia)(in reference to *all* clinical research)	‘research that has merit is justifiable by its potential benefit, which may include its contribution to knowledge and understanding, to improved social welfare and individual well-being, and to the skill and expertise of researchers’^21^
PHREB (Philippines)(in reference to *all* clinical research)	‘participation of human beings in research can only be justified if the study has social value’^24^
ICMR (India)(in reference to *all* clinical research)	‘research on human participants [is] … aimed at developing generalisable knowledge that improves health, increases understanding of disease and is ethically justified by its social value’^26^
MoST (Ethiopia)(in reference to *all* clinical research)	‘enhance health or generalisable knowledge, and benefit individuals and the community where the research is conducted’^27^
UNCST (Uganda)(in reference to *all* clinical research)	‘demonstrate value in terms of new knowledge added and probably improvement in health care provision and general social wellbeing’^29^
**2b. Balancing potential risks to participants against anticipated social value (‘risk-value’ assessment**)	
Tri-Council (Canada)	‘the nature of the research may justify some alteration(s) to consent requirements if the potential benefits outweigh the foreseeable risks’^19^
NHMRC (Australia)	‘the benefits from the research justify any risks of harm associated with not seeking consent’^21^
European Parliament & Council (EU) (in reference to cluster randomised trials only)	‘the protocol justifies the reasons for obtaining informed consent with simplified means’^30^

3. No more than minimal risk	
**3a. Minimal risk**	
CIOMS (International)	‘the research poses no more than minimal risks to participants’^18^
CFR (USA)	‘the research involves no more than minimal risk to the subjects’^17^
Tri-Council (Canada)	‘the research involves no more than minimal risk to the subjects’^19^
European Parliament & Council (EU) (in reference to cluster randomised trials only)	‘low-intervention clinical trials’ which ‘do not pose more than minimal additional risk or burden to the safety of the subjects compared to normal clinical practice’^30^
HRA (UK)	‘the study involves little or no deviation from usual care’ and ‘research risks are no greater than those involved in standard care/not greater than minimal’^20^
NHMRC (Australia)	‘involvement in the research carries no more than low risk’^21^
BAC (Singapore)	‘the research … poses no more than minimal risk to research participants’^22^
MREC (Malaysia)	‘subjects would be exposed to no more than minimal risk’^23^
PHREB (Philippines)	‘the research represents no more than minimal risk’^24^
MHLW (Japan)	‘the research to be implemented does not involve invasiveness (not including minor invasiveness)’^25^
ICMR (India)	‘the research involves less than minimal risk to participants’^26^
MoST (Ethiopia)	‘the research project carries no more than minimal risk’^27^
NHSRC (Malawi)	‘the research presents no more than a minimal risk of harm to the subjects’^28^
UNCST (Uganda)	‘the research project carries no more than minimal risk, that is, risk that is no more than the risks encountered in normal daily life in a stable society’^29^
**3b. Interventions available within current practice**	
European Parliament & Council (EU) (in reference to cluster randomised trials only)	‘there are no interventions other than the standard treatment of the subjects concerned’ and ‘the investigational medicinal products are used in accordance with the terms of the marketing authorisation’^30^
HRA (UK)	‘the study addresses a clinical question where there is uncertainty regarding the relative merits of relevant interventions’, ‘all medicines used in the trial are in routine use and within the terms of licence’ and ‘all interventions/diagnostic tests are in routine use within the NHS’^20^

4. Protection of welfare and rights	
CFR (USA)	‘the waiver or alteration will not adversely affect the rights or welfare of the subjects’^17^
Tri-Council (Canada)	‘alteration to consent requirements [must be] unlikely to adversely affect the welfare of participants’^19^
HRA (UK)	‘use of simplified means to obtain consent does not adversely affect the rights or welfare of study participants’^20^
BAC (Singapore)	‘the waiver will not adversely affect the welfare and interests of research participants’^22^
PHREB (Philippines)	‘the waiver or alteration will not adversely affect the rights and welfare of the participants’^24^
ICMR (India)	‘the waiver will not adversely affect the rights and welfare of the participants’^26^
MHLW (Japan)	‘the omission of procedures … is not against the research subjects’ interests’^25^
NHSRC (Malawi)	‘waiver is consistent with individual’s rights’^28^
HRA (UK)	‘patient has not expressed a strong preference for any particular treatment’^20^
NHMRC (Australia)	‘there is no known or likely reason for thinking that participants would not have consented’^21^

5. Protection of privacy and confidentiality	
NHMRC (Australia)	‘there is sufficient protection of (participants’) privacy’ and that ‘there is an adequate plan to protect the confidentiality of the data’^21^
BAC (Singapore)	‘individual privacy and confidentiality of the personal information are assured’^22^

6. Provision of information	
**6a. Provision of information after research participation**	
CFR (USA)	‘whenever appropriate, the subjects will be provided with additional pertinent information after participation’^38^
Tri-Council (Canada)	‘debriefing must be part of all research involving an alteration to consent… whenever it is possible, practicable and appropriate’ and ‘participants in such research must have the opportunity to refuse consent and request withdrawal of their data and/or human biological materials whenever possible, practicable and appropriate’^19^
European Parliament & Council (EU) (in reference to cluster randomised trials only)	‘describes the scope of information provided to the subjects, as well as the ways of providing information’^30^
NHMRC (Australia)	‘there is, where practicable, a plan for making information arising from the research available to [participants)’^21^
PHREB (Philippines)	‘participants will be provided with additional pertinent information after their participation (whenever appropriate)’^24^
MHLW (Japan)	‘to offer an ex-post explanation to the research subject etc. promptly’^25^
MoST (Ethiopia)	‘whenever appropriate, the research participants will be provided with additional pertinent information after participation’^27^
NHSRC (Malawi)	‘in lieu of a signed consent form, the NHSRC may require the investigator to provide subjects with a written statement regarding the research’^28^
UNCST (Uganda)	‘whenever appropriate the research participants will be provided with additional pertinent information after participation’^29^
**6b. Involvement of the community before research participation**	
Tri-Council (Canada)	‘researchers should … seek community engagement prior to data collection’^19^
MHLW (Japan)	'make announcement for the population to which the research subjects etc. belong’^25^
MoST (Ethiopia)	‘it is the responsibility of the investigator and the sponsor to publicise such a study to the community before commencement’^27^

7. Legal and financial obligations	
European Parliament & Council (EU) (in reference to cluster randomised trials only)	‘the simplified means for obtaining informed consent [does] not contradict national law in the Member State concerned’^30^
NHMRC (Australia)	‘the waiver is not prohibited by State, federal or international law’ and ‘the possibility of commercial exploitation of derivatives of the data or tissue will not deprive the participants of any financial benefits to which they would be entitled’^21^

BACBioethics Advisory CommitteeCIOMSCouncil for International Organisations of Medical SciencesHRAHealth Research AuthorityICMRIndian Council of Medical ResearchMHLWMinistry of Health, Labour and WelfareMoSTMinistry of Science and TechnologyMRECMedical Review & Ethics CommitteeNHMRCNational Health and Medical Research CouncilNHSRCNational Health Sciences Research CommitteePHREBPhilippine Health Research Ethics BoardUNCSTNational Council for Science and Technology

### Important social value and ‘risk-value’ assessment

The ‘important social value’ criterion is founded on beneficence ideals, with justification for carrying out the research being for the benefit of subjects, the community and society at large.^35 36^

This criterion is included, though variably worded, in several guidelines (table 3).^18 19 22 25^ In guidelines without a specific social value criterion for waiver of consent, social value is often stipulated as a broader requirement of *all* clinical research.^17 21 24 26 27 29 30^ Linked to this social value criterion is also the concept of balancing potential risks to participants against anticipated social value (‘risk-value’ assessment) which is expressed in some guidelines.^19 21 30 35^

### No more than minimal risk

The requirement of ‘no more than minimal risk’ aims to uphold non-maleficence principles.^31^ ‘Minimal risk’ is specifically defined in 12 guidelines, with unifying concepts being that the anticipated *probability* and *magnitude* of harm is small. The concept is sometimes expressed as no or minimal *additional* risk from participation in the research relative to that of not participating (the background risk of the management of the condition in clinical care).^1719^,^27 29 30^

The ‘no more than minimal risk’ criterion is contained within all guidelines examined (table 3).^17^,^30^ Four of the guidelines explicitly define ‘minimal risk’ as relating to both interventions/treatments *and* any associated tests/monitoring from conducting the research.^20 26 28 30^ Two of the guidelines explicitly refer to the testing of treatments currently available within clinical practice.^20 30^ The UK guidance document also comments on individual equipoise within cluster-determined interventions, stating that ‘healthcare professionals have the option of using an intervention other than the one assigned if they believe doing so is important for a particular patient’, relating to situations where clinician judgement deems neither clinical equipoise nor minimal risk apply.^20^

### Protection of welfare and rights

The ‘protection of participant welfare’ criterion closely relates to ‘minimal risk’ and broader principles of non-maleficence.^31^ ‘Welfare’ is discussed in seven guidelines (table 3).^1719 20 22 24^,^26^

The condition of a subject’s ‘rights’ not being adversely affected by the research is complex and raises the question of *which* rights are most relevant. Respect for individual autonomy may be thought to include the right to participate in research, choose between interventions and be informed throughout.^35^ However, Chalmers and Truog argue standard clinical care includes many low-risk decisions in which patients are neither involved in nor formally consented.^11 37^ Comparative effectiveness research and cluster randomised trials are frequently conducted in these usual care settings, and hence, it may be argued that participation need not involve any additional level of disclosure and consent.^31^ Acknowledging these contentions, the term ‘rights’ is detailed in the USA, UK, Philippines and Indian guidelines in conjunction with participant welfare^20 24 26 38^ and in the Malawian NHSRC guidelines which stipulate the ‘waiver is consistent with individual’s rights’.^28^ Furthermore, the UK draft guidance document requires that the ‘patient has not expressed a strong preference for any particular treatment’,^20^ and Australian NHMRC guidelines stipulate ‘there is no known or likely reason for thinking that participants would not have consented’,^21^ both of which pertain to maintenance of autonomy and individual rights.

### Protection of privacy and confidentiality

Maintaining patient privacy and confidentiality is an expected feature of all clinical research.^18^ This is specifically detailed in two waiver of consent guidelines (table 3).^21 22^

### Provision of information

Provision of pertinent information after research participation is founded on the ethical principle of respect,^39^ and it is detailed in nine guideline documents (table 3).^1921 24 25 27^,^30 38^ Provision of such information after participation acknowledges that while these low-risk trials may have scientific justification for waiver of consent at the time of trial conduct, that patients remain the primary stakeholders and there is a responsibility to maintain public confidence and accessibility of the research process.

Several documents also discuss the provision of information on a community level prior to conducting the research, including Canadian Tri-Council guidelines which, in relation to population and public health research, state that ‘researchers should … seek community engagement prior to data collection’,^19^ Japanese MHLW guidelines state that measures be taken ‘to make announcement for the population to which the research subjects etc. belong’,^25^ and Ethiopian MoST guidelines state that ‘it is the responsibility of the investigator and the sponsor to publicise such a study to the community before commencement’.^27^

### Legal and financial obligations

Reference to legal obligations, which closely relates to the principles of beneficence and non-maleficence, is discussed in two guidelines (table 3).^21 30^ Australian NHMRC guidelines also discuss financial obligations in reference to waiver of consent research, requiring that ‘the possibility of commercial exploitation of derivatives of the data or tissue will not deprive the participants of any financial benefits to which they would be entitled’.^21^

## Implications of waiver of consent policy

The individual ethical principles represented within contemporary intentional, regional and national guidelines regarding criteria for the use of waiver of consent in low-risk clinical research indicate several unifying concordances of waiver of consent policy. Collation of these guidelines in this review demonstrates overarching inclusion of the three central criteria put forward in the CIOMS guidelines for waiver of consent; that the research would not otherwise be feasible, the research has important social value and the research is minimal risk. Additional criteria contained within national and regional guidelines aim to provide further protection of research participants while enabling low-risk routine care studies that could not otherwise be conducted.

The CIOMS guidelines and their adoption within national/regional guidelines provide a framework for understanding situations where waiver of consent may be appropriate in study design. Importantly, these guidelines are not designed to alleviate researcher inconveniences; rather, they aim to provide a model for conducting ethically founded research and improving health that would otherwise not occur if other, more traditional, consent models are required.^34 37^ In the face of uncertainty and variations in practice, requiring a higher threshold for conducting a formal evaluation of treatments in RCTs than for making clinical treatment decisions will result in a paucity of evidence-based practice with no gain for patient autonomy. An equally important consideration is that in situations of true clinical equipoise, where practice variability lacks consensus rather than reflecting good individualised decision-making, preventing research that could not otherwise be carried out without a waiver of consent will lead to continued treatment variability that poses at least the same risk to current and future patients as random treatment assignment.^40^

Historically, participants in RCTs across a variety of specialities are poorly representative of the real-world patient population for which the intervention is intended, and this reflects strict eligibility criteria, volunteer bias and likely other biases.^4^,^7^ These are typically trials of novel agents where the effects of the agents on human health, including risks, are largely unknown. By contrast, low-risk pragmatic RCTs and cluster randomised studies are conducted in situations where all patients already receive one of several potential treatments, the relative benefits and risks of which are uncertain but the decision on which treatment to use is not based on high-quality, direct evidence. These randomised studies can use broad inclusion criteria, reflect real-world clinical situations and generate evidence clearly generalisable to a wider patient population.^41^ Waiver of consent is of value in these situations where the research would not otherwise be feasible for methodological reasons (eg, interventions with cluster-level participation or cluster-level outcomes), where there is no or minimal risk (with ‘minimal’ referring to both the severity and the probability of risk), and where there is anticipated to be significant individual, community and/or societal value and real-world evidence from the research being conducted. Anchoring expectations of consent between clinical settings and corresponding real-world research settings and recognising the role that waiver of consent holds in conducting such low-risk research that would not otherwise be feasible is fundamental to broadening pragmatic clinical research and the evaluation of real-world healthcare interventions. Accordingly, clinical research involving waiver of consent is gaining increasing awareness and has been successfully and appropriately used in a number of clinical studies (table 4).

**Table 4:** Examples of completed clinical research studies using waiver of informed consent

Study name	Study characteristics	Primary indication(s) for waiver of consent	Study impact
*Personalised cooler dialysate for patients receiving maintenance haemodialysis (MyTEMP): a pragmatic, cluster-randomised trial* ^44^	Large pragmatic, cluster-randomised trial conducted in haemodialysis centres in Canada, comparing standard temperature dialysate (36.5°C) vs personalised cooler dialysate temperature (0.5°C below the patient’s body temperature with a minimum of 35.5°C and maximum of 36.5°C) among patients receiving maintenance haemodialysis	A minimal risk study The intervention compared two low-risk practice variations Randomisation occurred at the cluster level (ie, haemodialysis centres were randomised as clusters) and attempts to consent individuals would lead to contamination and severe bias in the study design	4.3 million haemodialysis treatments were provided to 15 413 patients across 84 haemodialysis centres in this large pragmatic trial, which demonstrated no difference in major adverse cardiovascular events or intradialytic hypotension events between the intervention arms
*Comparative effectiveness of high-dose vs standard-dose influenza vaccination on numbers of US nursing home residents admitted to hospital: a cluster-randomised trial* ^45 46^	Large pragmatic, comparative effectiveness, cluster-randomised trial compared high-dose vs standard-dose influenza vaccination in nursing home residents in the USA to assess the effect on hospitalisations, mortality and functional decline	A minimal risk study A population at high risk of the outcome being assessed who are typically poorly represented in traditional randomised controlled trials Randomisation occurred at the cluster level (ie, nursing homes were randomised as clusters), and attempts to consent individuals would lead to contamination and severe bias in the study design	823 nursing homes with 92 269 residents were included in this large and generalisable trial, which demonstrated that high-dose influenza vaccination reduces the risk of respiratory-related hospital admissions for nursing home residents
*Comparative effectiveness of a multifaceted intervention to improve adherence to annual colorectal cancer screening in community health centres: A randomised clinical trial* ^47 48^	Patient-level randomised controlled trial of patients in participating community health centres in the USA who were identified from electronic health records and randomised to receive either usual care for colorectal cancer screening (computerised reminders to healthcare providers and clinician feedback on screening rates) or a multifaceted intervention to facilitate colorectal cancer screening (mailed reminder letter to patients, mail out of a home faecal occult blood test kit, telephone and text message reminders to patients)	A minimal risk study Informed consent is not scientifically feasible without compromising the validity of the study in delivering an intervention to improve screening adherence Assessing a fully representative population (including low socioeconomic groups and ethnic minority groups who are typically poorly represented in randomised controlled trials involving traditional consent models) All patient information and outcome data collection were obtained from routine care records	450 patients were identified and randomised to the two arms, with the low-cost multifaceted intervention strategy achieving a much higher rate of adherence to colorectal cancer screening (82.2%) compared with the usual care group (37.3%)

Ethics committees can provide valuable oversight in research conducted under a waiver of consent model. Well-resourced and well-informed expert ethics committees that are familiar with the ethical considerations for waiver of consent studies are best placed to protect the safety of participants. Oversight of data security frameworks is a key consideration in research conducted with a consent waiver. Human research ethics paradigms evolved to provide community oversight of and trust in medical research.^42^ Adherence to the highest standards in the conduct and oversight of waiver of consent studies is essential to maintaining public trust. Some statements explicitly suggest contemporaneous publication of waiver of consent approvals as a way of informing the community at large of research that involves individuals within that community.^21^ The role of guidance such as that presented in the current collation of waiver of consent statements is a step towards facilitating appropriate implementation of ethical principles and a common high standard in the conduct of waiver of consent research.

### Strengths and limitations

International, regional and national ethical statements and guidelines on waiver of consent in clinical research historically have been presented in various online formats and have not been available in peer-reviewed published literature. This review collates a broad range of guidelines to provide researchers and ethics committees alike with a summary of principles that were deemed requirements for considering a waiver of consent model for any individual study. They support the design, oversight and conduct of collaborative multicentre international research that may warrant a waiver of consent model. However, there are important limitations to this review that should be considered. First, international, regional and national ethical statements and guidelines on waiver of consent in clinical research have been presented in various online formats and have not been available in peer-reviewed published literature. Although a detailed search was employed, it is possible some relevant documents may have been missed. Second, our inclusion criteria were restricted to English-language documents. Finally, ethical guidelines are subject to review and individual statements may be revised or modified over time. A dynamic online platform which collates real-time recommendations and guidance on human research ethical issues (including waiver of consent) would be worthy of consideration to assist researchers and ethics committees and enhance engagement from the broader community.

## Conclusions

National, regional and international frameworks exist for the utilisation of waiver of consent in low-risk clinical research studies that closely resemble usual practice under the oversight of designated ethical bodies. This paper provides a summary of international approaches to waiver of consent research together with the level of consensus for the individual principles. These statements have a unifying basis consistent with the 2016 CIOMS guidelines with three overarching criteria: that the research would not otherwise be feasible, the research has important social value and the research is minimal risk. Additional criteria contained within some but not all national and regional guidelines aim to provide additional and appropriate protection to research participants. The guidelines collated in this review provide researchers with a clear and harmonised framework for conducting low-risk pragmatic clinical research that would not otherwise be feasible, through rigorous assessments of the relative effectiveness of variations in clinical practice, identification of optimal practices and, ultimately, leading to improved healthcare outcomes.
