# Supplementary material for: Waiver of informed consent in clinical research: a summary of contemporary guidelines and a resource for researchers
Source: BMJ Open. 2025 Mar 18;15(3):e091896. doi: 10.1136/bmjopen-2024-091896 (PMC11927427; doi:10.1136/bmjopen-2024-091896)
Supplement: Supplementary file 1 [file bmjopen-2024-091896.reviewer_comments.pdf]

PEER REVIEW HISTORY

BMJ Open publishes all reviews undertaken for accepted manuscripts. Reviewers are asked to complete a checklist review form and are provided with free text boxes to elaborate on their assessment. These free text comments are reproduced below.

ARTICLE DETAILS

Title (Provisional)

Waiver of informed consent in clinical research: A summary of contemporary guidelines & a resource for researchers

Authors

Siriwardana, Amanda; Smyth, Brendan; Jardine, Meg

VERSION 1 - REVIEW

**Reviewer**	**1**
**Name**	**Shi, Xiaoting**
**Affiliation**	**Yale University School of Public Health, Department of Environmental Health Sciences**
**Date**	**08-Oct-2024**
**COI**	**None**

Thank you for the opportunity to review this manuscript. In this manuscript by Dr. Siriwardana et al., the authors recorded international, regional and national guidelines on waiver of consent in clinical research. Extracting key information from 14 guidelines, the authors summarized the principles in each for circumstances in which a waiver is appropriate. Overall, the authors identified seven principles and discussed them in detail in the manuscript. This is an important and interesting topic, as it is a good starting point to help address the barrier for certain clinical research. The tables and figure clearly and in detail showed where the principles came from, which were helpful to the readers. However, there are several aspects where additional clarity is necessary:

First, throughout the manuscript, the authors did not provide a clear description of their methods, which makes the results hard to reproduce. In particular, how were the 14 guidelines identified and selected? What database/websites were searched? What were the criterion of including these guidelines (e.g., what keywords/section(s) are required to include)?

Accordingly, a Limitation section outlining the limitations of the methods that they used is also required. It is also recommended to discuss the potential limitations/threats of waiving informed consents in clinical research in this section or in the Discussion section.

Moreover, the discussion section now is a bit thin, which is now under the section named Implications of waiver of consent policy. The authors could consider adding the potential next steps for future research to consider. For example, what criterion are needed to consider a waiver of informed consent and how or why are these selected.

Lastly, in the manuscript, the authors briefly touched upon in what situations waiver of consent is of most value. If possible, the authors could expand on this section and talk a bit more in order to inform different stakeholders.

Reviewer **2**

Name **Jamrozik, Euzebiusz**

Affiliation **University of Oxford, Ethox and the Wellcome Centre for Ethics and Humanities**

Date **11-Oct-2024**

COI **None**

This paper provides an excellent summary of waiver of consent requirements. The sections devoted to this review would warrant publication as a standalone paper focused on this summary.

However, the abstract, introduction, and discussion are excessively focused on a single area of research (viz. "learning health system environments"), and this is not the only non-emergency research area where waiver of consent is relevant.

I would encourage the authors to rewrite these sections to focus on what I take to be the central goal of the paper, i.e., to summarise waiver of consent policies. The discussion should then give a more balanced summary of (i) how these waiver policies might be relevant to a wider variety of non-emergency research (including but not limited to learning health system environments), (ii) why these waivers might not more widely recognised or implemented, etc.

One of the interesting points about minimal risk here, including as it is relevant to learning health systems, is what the relevant baseline should be. Health systems with wide variations in practice arguably do involve significant risk (related to practice variation) where patients are not aware of this risk or the alternative possible treatment options. On one view, randomizing patients in research comparing these different practice variations arguably involves minimal or no risk compared to baseline (if the relevant baseline is that patients are randomly admitted under different doctors with different practices). On another view, once it becomes research, the risks of different practices (now different arms of the trial) are ethically significant, because the baseline risk is perceived to be that of the person entering the health system. For a good introduction to this area of debate, see this paper: https://link.springer.com/article/10.1007/s40592-014-0007-6

Space permitting, spending more of the discussion on similar analyses of points 1-7 in the review and how these might play out in the design, ethical review, and conduct of research would be valuable - rather than focusing only on learning health systems.

Alternatively, the authors could change the title and focus of the paper to be only about how waiver of consent can facilitate learning health system environments. But

this would be a different paper - and lose the benefit of having a good review of waiver of consent more generally.

**Reviewer**	**3**
**Name**	**Ribeiro, Maria Alexandra**
**Affiliation**	**Universidade Nova de Lisboa**
**Date**	**21-Oct-2024**
**COI**	**None**

Comments to the Author

The authors provided a clear and systematic overview of the scope of "waiver of consent" in the guidelines used in this paper (Table 1) and report the presence/absence of criterion for waiver of consent in each guideline used (Table 2). Both tables are useful from the pragmatic point of view reflecting the contents of each reviewed guideline on the waiver of informed consent topic. Hower the paper reveals some weaknesses. To improve the paper and make it recommended for publication some clarifications should be made and/or information should be added to the text.

1) It is not clear/ understandable why authors refer the objective of this guidelines review "specifically" on the category of "non-emergency clinical studies".
    a. Are, for instance, guidelines on clinical trials (with medicines) or clinical investigations with Medical Devices outside emergency settings included in this revision of guidelines?
    b. The use of "low-risk" clinical studies´ category/expression is not fully understood.
    c. The rational under this study written in the abstract: "Given the growing importance of routine-care embedded research activity, we sought to describe….." (p. 3: lines 16-17) seems not to be related with "non-emergency clinical studies/research" category.
    d. Abstract and Introduction are not clearly aligned (coherent) regarding the focus and objective of the studies that were considered for the guidelines review that are described in this paper.
        - From abstract it seems that the revision of the guidelines was made for research (pragmatic studies) using data collected in routine clinical practice.
        - From the introduction it seems that the approach is broader: "all situations in which investigators are not required to ….participation(16). Specifically, ….guidelines for non-emergency research…" (p. 5 : line 60 and p.6:lines 3-6)

2) It is not justified, nor is it mentioned in the text how and why the guidelines used in the review procedure were identified. Are they the only existing ones? What were the criteria for using these? Are these a convenience sample? Despite not being a systematic review some information on the chosen guidelines should be made. In this line no limitations of the study are identified or mentioned.

3) It is not comprehensive (nor described) why authors use CIOMS Guideline as the "reference" Guideline in the paper. For example:
   - In the Section "INTERNATIONAL, REGIONAL AND NATIONAL GUIDELINES ON WAIVER OF CONSENT" describes the 3 essential criteria (p. 27) and "just" refers to the other criteria and guidelines in the corresponding table.
   - For the conclusions (conclusion Section) authors "compare" the other guidelines framework for using waiver of consent with "reference" CIOMS Guideline".
   - Being somehow understandable for a knowledgeable reader in this topic (clinical research and waiver of consent) the paper should reflect why CIOMS guideline is the reference.

4) Conclusion section: There are no other conclusions (outcomes) from the analysis of the guidelines besides comparing them with the CIOMS guideline? This consideration is also related with the consideration number 3) above. The conclusion section should be improved, unless authors consider the objective of the study comparing the several guidelines reviewed with the CIOMS Guideline. If not other outcomes of the study should be identified.

5) References: No references since 2021 were included despite several important papers were published on the paper´s topic (some important to consider). Authors are advised to make a review of recent papers and consider the possibility of including them.

6) Other minor corrections should be made:
   - The European Union guideline identified as the regional guideline in the abstract is no longer mentioned on paper (unless in both describing tables). On the other hand, the CIOM´s guideline used as the "reference" is not mentioned as the international Guideline in the abstract. Please add the CIOMS guideline in the Abstract section as the regional one (line 22).
   - Rephrase the period "in collaboration with…..2002 guideline" starting with "The international Ethical guideline…." to best understand the importance – internationality-of this guideline. (Why using this guideline as the reference and its importance might be included in this first paragraph of this section or as a second paragraph and then refer to the "three essential criteria".

**Reviewer**	**4**
**Name**	**James, John T.**
**Affiliation**	**Patient Safety Amer, CEO**
**Date**	**29-Oct-2024**
**COI**	**None**

The authors show that the guidelines from various sources differ in their content when addressing the need for clinical studies in which informed consent from the patient should be waived. The primary guideline source originated from the WHO in 2016 and requires three essential criteria be met: informed consent would not be feasible or practical, the proposed research has important social value, and the research poses no more than minimal risk. My italicized words require subjective ethical judgment on the part of a clinical research governing body and the clinical researchers. The authors attempt to better define each of the subjective areas, and then add four more pertinent declarations from other sources of guidelines.

While the compilation of guidelines is interesting, it is unclear to me how all this adds to patient safety except in the few cases where waiver of consent can be ethically applied to research that leads to improved health that could not be discerned in any other way. In that regard, it would greatly improve the value of the manuscript if the following were addressed: (1) describe several specific examples of clinical research that received a waiver of informed consent and fully met the three criteria in the WHO document. The 'declaration' of minimal risk requires further discussion. Risk is a combination of severity of harm and probability of that harm. (2) How do severity and probability work together to define 'minimal' risk to patients in the real world of clinical medicine. The authors rightly conclude that waiver of informed consent research may 'facilitate learning health system environments that can provide rigorous assessments of the relative effectiveness of variations in clinical practice.' I am well aware that there are important variations in clinical practice; however, this begs the question of generalizability of findings from studies conducted on limited cohorts with patients that did not receive informed consent. (3) Specific examples of how this has been applied in the real world to facilitate learning among health systems would strengthen the manuscript. (4) How has informed-consent-waived research resulted in broad scale learning outside the 'system' where the research was conducted. (5) How has a waiver-of-informed-consent study led to changes in medical guidelines?

In the end, the study, as it is, belongs in a journal of ethics. However, if the practical applications can be shown in specific medical situations, then it may be appropriate for the BMJ.

VERSION 1 - AUTHOR RESPONSE

REVIEWER 1

How were the 14 guidelines identified and selected? What database/websites were searched? What were the criterion of including these guidelines (e.g., what keywords/section(s) are required to include)?

Response: We employed an extensive online search to collate national/regional/international statements into a useable and published reference paper for future researchers and ethics committees alike. We have described the methods used in two dedicated Methods paragraphs on page 5 of the manuscript:

'This analysis examines current international, regional and national English-language guidelines on 'waiver of consent' in clinical research within non-emergency settings. 'Waiver of consent' was defined as situations where investigators are not required to inform or request permission from research subjects prior to participation (17). Emergency care research was excluded as such research involves critically unwell individuals who are unable to consent and/or involve time-critical emergency interventions, for which modified or delayed consent is typically employed (ie. a temporary waiver of consent followed by seeking consent at a later date once the patient is stable, or from an authorised representative or guardian). The ethical principles relevant to such situations are distinct from that of waiver of consent in non-emergency clinical research.

We included all available English-language ethical guidelines or statements on the use of waiver of consent for non-emergency clinical research. We searched the bibliographic databases MEDLINE and EMBASE using the search terms: 'research' or 'clinical research' or 'medical research' or 'health research', and 'consent' or 'informed consent' or 'ethics' or 'medical ethics', and 'waiver'. We also conducted an extensive online search for 'grey literature' using the above terms on the rationale that documents relevant to this review may be contained within health research regulatory guidelines produced by government agencies and not published in peer-reviewed literature.'

A limitation section outlining the limitations of the methods that they used is also required.

Response: A 'Strengths & Limitations' section has been added to page 11 of the manuscript following the discussion section. To discuss the limitations of our methodology and manuscript, the following text has been added:
'This review collates a broad range of guidelines to provide researchers and ethics committees alike with a summary of principles that were deemed requirements for considering a waiver of consent model for any individual study. They support the design, oversight and conduct of collaborative multi-centre international research that may warrant a waiver of consent model. However, there are important limitations to this review that should be considered. Firstly, international, regional and national ethical statements and guidelines on waiver of consent in clinical research have been presented in various online formats and have not been available in peer-reviewed published literature. Although a detailed search was employed, it is possible some relevant documents may have been missed. Secondly, our inclusion criteria were restricted to English-language documents. Finally, ethical guidelines are subject to review and individual statements may be revised or modified over time.'

It is also recommended to discuss the potential limitations/threats of waiving informed consents in clinical research in this section or in the Discussion section.

Response: We have made a number of additions to the manuscript to delineate the limited and clear situations for waiving informed consent. Firstly, our paper is founded in a position of agreement with the reviewer's underlying sentiment that the appropriateness of waiver of consent is situationally specific. In our revised version of the paper, the introduction distinguishes situations where a waiver may be appropriate in contrast to situations where it is not (eg. the reference to '*research involving novel therapies and technologies*', the contrast of waiver situations with the default scenario of '*individual randomised controlled trial designs*', and '*traditional informed consent models*', the careful description of learning health system research within '*areas of existing variation in clinical practice that are not justified by high-quality evidence or local conditions*'). The introduction refers to the difference between '*explanatory' RCTs*' and '*comparative effectiveness studies*'. It suggests an anchoring to consent practices in clinical practice ('*such studies of practices where patient consent is not sought in clinical practice <u>may</u> warrant a similar waiver of research consent…').*

Our purpose in writing this paper was to readily equip researchers, ethics committees and the health community with a distillation of global guidance on when waiver of consent is appropriate, built on the implicit assumption that informed consent is the default approach.

We have made this purpose more explicit and added the following to the outline of the aims of the paper in the Introduction on page 5:
'The increasing recognition of the impact of health services on clinical outcomes comes at a time when digital advancements are facilitating research and quality assurance studies that were previously technologically challenging or impossible (2). In this context there is increasing interest in the utilisation of waiver of consent in low-risk clinical research. While the well-established opt-in consent model has broadly accepted advantages for participant autonomy and safety, researchers, ethics committees and the broader health community may benefit from a distillation of global guidance on situations in which it may be ethically acceptable to consider a waiver of consent. We therefore aimed to provide an overview of national and international statements of guidance on principles that outline when a waiver of consent model could be considered for an individual study.'

We have also added the following paragraph to the Discussion section on page 10:
'Ethics committees can provide valuable oversight in research conducted under a waiver of consent model. Well-resourced and well-informed expert ethics committees that are familiar with the ethical considerations for waiver of consent studies are best placed to protect the safety of participants. Oversight of data security frameworks is a key consideration in research conducted with a consent waiver. Human research ethics paradigms evolved to provide community oversight of and trust in medical research (42). Adherence to the highest standards in the conduct and oversight of waiver of consent studies is essential to maintaining public trust. Some statements explicitly suggest contemporaneous publication of waiver of consent approvals as a way of informing the community at large of research that involve individuals within that community. The role of guidance such as that presented in the current collation of waiver of consent statements is a step towards facilitating appropriate implementation of ethical principles and a common high standard in the conduct of waiver of consent research.'

The authors could consider adding the potential next steps for future research to consider. For example, what criterion are needed to consider a waiver of informed consent and how or why are these selected.

Response: The manuscript systematically identifies and collates criterion considered necessary for waiver of consent developed independently by national and international bodies. We further describe the 7 individual criteria that were common across waiver of consent guidance statements which are summarised in *Table 2* and *Table 3*. The manuscript provides a summary of international approaches to waiver of consent research together with

the level of consensus for the individual principles. We believe a strength of our approach is that we are not presenting our individual views but rather are distilling the principles of multiple guidance documents prepared independently by national and international bodies. We agree that there is always a role for further work in research-critical domains. In this case, we feel that a mechanism to maintaining currency is a key issue through a mechanism such as the collation of guidelines in a real-time platform. We have therefore added the following sentences to page 11: *'…ethical guidelines are subject to review and individual statements may be revised or modified over time. A dynamic online platform which collates real-time recommendations and guidance on human research ethical issues (including waiver of consent) would be worthy of consideration to assist researchers and ethics committees and enhance engagement from the broader community.'*

The authors briefly touched upon in what situations waiver of consent is of most value. If possible, the authors could expand on this section and talk a bit more in order to inform different stakeholders.

Response: As discussed further below in response to Reviewer 2's comments, we have made amendments to the Title, Abstract, Introduction and Discussion to more clearly emphasise the role of this paper (to summarise waiver of consent policies) and the situations in which it may be of value. In particular, we have aimed to better emphasise the applicability of waiver of consent to low-risk clinical research more broadly, which includes but is not limited to learning health system research.

REVIEWER 2

The abstract, introduction, and discussion are excessively focused on a single area of research (viz. "learning health system environments"), and this is not the only non-emergency research area where waiver of consent is relevant.

I would encourage the authors to rewrite these sections to focus on what I take to be the central goal of the paper, i.e., to summarise waiver of consent policies. The discussion should then give a more balanced summary of (i) how these waiver policies might be relevant to a wider variety of non-emergency research (including but not limited to learning health system environments), (ii) why these waivers might not more widely recognised or implemented, etc.

Space permitting, spending more of the discussion on similar analyses of points 1-7 in the review and how these might play out in the design, ethical review, and conduct of research would be valuable - rather than focusing only on learning health systems.

Response: The authors thank Reviewer 2 for highlighting the broader context of our manuscript beyond learning health system research. Accordingly, we have made edits and additions to the Title, Abstract, Introduction and Discussion to emphasise the broader applicability of waiver of consent to low-risk clinical research, including but not limited to learning health system environments. With the edits of these sections, we have aimed to emphasise the primary goal of this paper which is to summarise the waiver of consent guidelines/statements into a useable reference piece that informs research practice in this area. The following edits have been made:

- Change to Manuscript Title: *'Waiver of informed consent in clinical research: A summary of contemporary guidelines & a resource for researchers'.*
- Additions to the opening lines of the Abstract on page 2 have been made: *'Low-risk pragmatic clinical research such as learning health system research, quality assurance activities and comparative effectiveness studies are approaches of embedding clinical research within routine practice to generate generalisable evidence. Individual written informed consent may present a barrier to the feasibility and inclusiveness of such low-risk clinical research…'*
- Extensive revisions to the first paragraph of the Introduction on page 4 which now reads as: *'Informed consent for research participation is a cornerstone of modern medical ethics and is an essential feature of randomised research involving novel therapies and technologies. While such studies spearhead progress in patient care, many aspects of the health care system are unsuited to individual randomised controlled trial designs or seek to assess the impact of differing treatment strategies or systems on broad patient populations that are often not fully captured by studies using traditional informed consent models. This is most relevant for pragmatic low-risk clinical research which aims to evaluate the effectiveness of interventions in real-world settings, often comparing the relative merits of medical treatments already in common use. Such research is often cited as key to achieving the goal of a 'learning health system', in which research is embedded within routine practice and the effectiveness of an intervention, practice or system change is assessed in all participants exposed (1). Comparative effectiveness pragmatic randomised controlled trials (RCTs) are a form of learning health system research, which act within areas of existing variation in clinical practice that are not justified by high-quality evidence or local conditions (2, 3). These studies replace arbitrary treatment*

selection with randomised selection and are essential to improving patient outcomes, especially with respect to institutional or health system practices that are determined outside of, or prior to, individual clinician-patient treatment decisions.'

- Additions to the 2nd paragraph of the Introduction on page 4 have been made to better place where waiver of consent has a role in low-risk clinical research: *'Exploratory randomised trial populations have historically differed substantially from the broader patient population (4-7). These differences stem in part from inclusion and exclusion criteria that are designed both to protect participant safety and to define a population most suited to testing the study hypothesis. However, they also result from the informed consent process itself, which requires participants understand consent material written in technical and legal style (or have a guardian able to do so), and to sufficiently engaged in their healthcare so as to volunteer or accept referral for inclusion in research. These factors result in study populations which differ in important ways from the broader patient population, for example being younger, less comorbid, or with a better prognosis. Contrastingly, comparative effectiveness studies and evaluations of health-service interventions are designed for broader real-world settings and will naturally lead to changes in practice that affect all patients. Such studies will be most informative if they include generalisable populations, are sufficiently large, and minimise bias. Given the barriers that traditional written informed consent models pose to inclusion of a representative patient population, such studies of practices where patient consent is not sought in clinical practice may warrant a similar waiver of research consent to minimise selection and other biases (8-10).'*
- An additional first paragraph has been added to the beginning of the Discussion on page 9 to summarise the applicability of the paper to low-risk clinical research more broadly: *'The individual ethical principles represented within contemporary intentional, regional and national guidelines regarding criteria for the use of waiver of consent in low-risk clinical research indicate several unifying concordances of waiver of consent policy. Collation of these guidelines in this review demonstrates overarching inclusion of the three central criteria put forward in the CIOMS guidelines for waiver of consent; that the research would not otherwise be feasible, the research has important social value, and the research is minimal risk. Additional criteria contained within national and regional guidelines aim to provide further protection of research participants while enabling low-risk routine-care studies that could not otherwise be conducted.'*
- As mentioned, an additional final paragraph has been added to the Discussion on page 10 to highlight some of the challenges/cautions with using waiver of consent in

clinical research: *'Ethics committees can provide valuable oversight in research conducted under a waiver of consent model. Well-resourced and well-informed expert ethics committees that are familiar with the ethical considerations for waiver of consent studies are best placed to protect the safety of participants. Oversight of data security frameworks is a key consideration in research conducted with a consent waiver. Human research ethics paradigms evolved to provide community oversight of and trust in medical research (42). Adherence to the highest standards in the conduct and oversight of waiver of consent studies is essential to maintaining public trust. Some statements explicitly suggest contemporaneous publication of waiver of consent approvals as a way of informing the community at large of research that involve individuals within that community (21). The role of guidance such as that presented in the current collation of waiver of consent statements is a step towards facilitating appropriate implementation of ethical principles and a common high standard in the conduct of waiver of consent research.'*

REVIEWER 3

1) It is not clear/ understandable why authors refer the objective of this guidelines review "specifically" on the category of "non-emergency clinical studies".

a. Are, for instance, guidelines on clinical trials (with medicines) or clinical investigations with Medical Devices outside emergency settings included in this revision of guidelines?

b. The use of "low-risk" clinical studies´ category/expression is not fully understood.

c. The rational under this study written in the abstract: "Given the growing importance of routine-care embedded research activity, we sought to describe….." (p. 3: lines 16-17) seems not to be related with "non-emergency clinical studies/research" category.

d. Abstract and Introduction are not clearly aligned (coherent) regarding the focus and objective of the studies that were considered for the guidelines review that are described in this paper.

- From abstract it seems that the revision of the guidelines was made for research (pragmatic studies) using data collected in routine clinical practice.

- From the introduction it seems that the approach is broader: "all situations in which investigators are not required to ….participation(16). Specifically, ….guidelines for non-emergency research…" (p. 5 : line 60 and p.6:lines 3-6)

Response: We thank Reviewer 3 for the comments regarding the scope of our paper and what is meant by low-risk clinical research. These echo Reviewer 2's comments regarding the applicability of waiver of consent considerations in multiple settings including learning health system research, other low-risk clinical research and (from this reviewer) emergency clinical studies. Please refer to the detailed response to Reviewer 2's comments and the amendments made to the Title, Abstract, Introduction and Discussion to more clearly define low-risk clinical research settings for which waiver of consent may be relevant.

We focussed our review on non-emergency care studies, due to differences in ethical principles and rationales for waiver of consent in the emergency and non-emergency settings. In accordance with international ethical guidelines, it has been our perception that modified or delayed consent in the emergency setting has broad acceptability. Emergency studies conducted with modified or delayed consent (ie. a temporary waiver of consent followed by seeking consent at a later date once the patient is stable, or from an authorised representative or guardian) are justified on the time-critical nature of many emergency interventions when potential participants are often critically unwell and unable to consent. The rationale for a waiver of consent approach when time was not limited requires additional consideration. We have added the following to the Methods section of the manuscript on page 4 to clarify this: *'Emergency care research was excluded as such research involves critically unwell individuals who are unable to consent and/or involve time-critical emergency interventions, for which modified or delayed consent is typically employed (ie. a temporary waiver of consent followed by seeking consent at a later date once the patient is stable, or from an authorised representative or guardian). The ethical principles relevant to such situations are distinct from that of waiver of consent in non-emergency clinical research.'*

2) It is not justified, nor is it mentioned in the text how and why the guidelines used in the review procedure were identified. Are they the only existing ones? What were the criteria for using these? Are these a convenience sample? Despite not being a systematic review some information on the chosen guidelines should be made. In this line no limitations of the study are identified or mentioned.

Response: As discussed above in response to Reviewer 1's comments, both Methods and Strengths & Limitations sections have now been added to the manuscript to more clearly explain these aspects for the reader.

The Methods section on page 5 is as follows:

‘This analysis examines current international, regional and national English-language guidelines on ‘waiver of consent’ in clinical research within non-emergency settings. ‘Waiver of consent’ was defined as situations where investigators are not required to inform or request permission from research subjects prior to participation (17). Emergency care research was excluded as such research involves critically unwell individuals who are unable to consent and/or involve time-critical emergency interventions, for which modified or delayed consent is typically employed (ie. a temporary waiver of consent followed by seeking consent at a later date once the patient is stable, or from an authorised representative or guardian). The ethical principles relevant to such situations are distinct from that of waiver of consent in non-emergency clinical research.

We included all available English-language ethical guidelines or statements on the use of waiver of consent for non-emergency clinical research. We searched bibliographic databases MEDLINE and EMBASE using the search terms: ‘research’ or ‘clinical research’ or ‘medical research’ or ‘health research’, and ‘consent’ or ‘informed consent’ or ‘ethics’ or ‘medical ethics’, and ‘waiver’ or ‘simplified’. We also conducted an extensive online search for ‘grey literature’ using the above terms on the rationale that documents relevant to this review may be contained within health research regulatory guidelines produced by government agencies and not published in peer-reviewed literature.’

The Strengths & Limitations section on page 11 is as follows:
‘International, regional and national ethical statements and guidelines on waiver of consent in clinical research historically have been presented in various online formats and have not been available in peer-reviewed published literature. This review collates a broad range of guidelines to provide researchers and ethics committees alike with a summary of principles that were deemed requirements for considering a waiver of consent model for any individual study. They support the design, oversight and conduct of collaborative multi-centre international research that may warrant a waiver of consent model. However, there are important limitations to this review that should be considered. Firstly, international, regional and national ethical statements and guidelines on waiver of consent in clinical research have been presented in various online formats and have not been available in peer-reviewed published literature. Although a detailed search was employed, it is possible some relevant documents may have been missed. Secondly, our inclusion criteria were restricted to English-language documents. Finally, ethical guidelines are subject to review and individual statements may be revised or modified over time. A dynamic online platform which collates real-time recommendations and guidance on human research ethical issues (including

waiver of consent) would be worthy of consideration to assist researchers and ethics committees and enhance engagement from the broader community.'

3) It is not comprehensive (nor described) why authors use CIOMS Guideline as the "reference" Guideline in the paper. For example:

- In the Section "INTERNATIONAL, REGIONAL AND NATIONAL GUIDELINES ON WAIVER OF CONSENT" describes the 3 essential criteria (p. 27) and "just" refers to the other criteria and guidelines in the corresponding table.

- For the conclusions (conclusion Section) authors "compare" the other guidelines framework for using waiver of consent with "reference" CIOMS Guideline".

- Being somehow understandable for a knowledgeable reader in this topic (clinical research and waiver of consent) the paper should reflect why CIOMS guideline is the reference.

Response: The CIOMS guideline is the primary international document pertaining to the conduct of health-related human research, originating from a collaboration with the World Health Organisation (WHO). It is widely considered the main reference and 'gold standard' document for ethical research conduct. As stated within the CIOMS guidelines and by the WHO, the aims of the CIOMS guidelines are to both reflect input from WHO member states and to then provide a global reference point for researchers and regulators in any jurisdiction worldwide. As such, we have elected treat the CIOMS guideline as the primary reference.

We have made additions to the first two paragraphs of the 'International, regional and national guidelines on waiver of consent' section of the manuscript on page 6 to better articulate this to the reader:

'Our search yielded 14 eligible reference documents. None were obtained through database searches of MEDLINE and EMBASE, and all were obtained from 'grey literature' sources. These statements included one international and 13 national/regional guidelines.

The International Ethical Guidelines for Health-related Research Involving Humans were first released in 1982 and have undergone several revisions, most recently in 2016 (18). These international guidelines are a collaboration between the Council for International Organisations of Medical Sciences (CIOMS) and the World Health Organisation (WHO), and provide a global framework of ethical principles to guide the conduct of all health-related research involving human participants. Guideline 10 pertains to waiver of consent. In addition to the CIOMS international guideline, we identified 1 regional (European Union) and

12 national guidelines applicable in 39 countries (Figure 1). These documents and their intended scope are detailed in Table 1.'

4) Conclusion section: There are no other conclusions (outcomes) from the analysis of the guidelines besides comparing them with the CIOMS guideline? This consideration is also related with the consideration number 3) above. The conclusion section should be improved, unless authors consider the objective of the study comparing the several guidelines reviewed with the CIOMS Guideline. If not other outcomes of the study should be identified.

Response: The primary aim of this manuscript was to collate ethical guidelines pertaining to waiver of consent, in order to guide researchers and ethics boards alike on how and where waiver of consent may be necessary to conduct ethically-sound low-risk clinical research. We have made additions to the Conclusions section on page 11 to better articulate the aims of the paper and how this is achieved:
'National, regional and international frameworks exist for the utilisation of waiver of consent in low-risk clinical research studies that closely resemble usual practice under the oversight of designated ethical bodies. This paper provides a summary of international approaches to waiver of consent research together with the level of consensus for the individual principles. These statements have a unifying basis consistent with the 2016 CIOMS guidelines with three overarching criteria: that the research would not otherwise be feasible, the research has important social value, and the research is minimal risk. Additional criteria contained within some but not all national and regional guidelines aim to provide further and appropriate protection to research participants. The guidelines collated in this review provide researchers with a clear and harmonised framework for conducting low-risk pragmatic clinical research that would not otherwise be feasible, through rigorous assessments of the relative effectiveness of variations in clinical practice, identification of optimal practices and, ultimately, leading to improved health care outcomes.'

5) References: No references since 2021 were included despite several important papers were published on the paper´s topic (some important to consider). Authors are advised to make a review of recent papers and consider the possibility of including them.

Response: The authors thank Reviewer 3's comments. We have updated the references (including references 32, 34, 42 and 44).

6) Other minor corrections should be made:

- The European Union guideline identified as the regional guideline in the abstract is no longer mentioned on paper (unless in both describing tables). On the other hand, the CIOM´s guideline used as the "reference" is not mentioned as the international Guideline in the abstract. Please add the CIOMS guideline in the Abstract section as the regional one (line 22).

Response: We have adopted the Reviewer's suggestion and made amendments to the Abstract on page 2, which now reads as: *'We identify 14 guidelines including 1 international, 1 regional and 12 national statements, and summarise the principles in each for circumstances in which a waiver of consent is appropriate.'*

- Rephrase the period "in collaboration with…..2002 guideline" starting with "The international Ethical guideline…." to best understand the importance – internationality-of this guideline. (Why using this guideline as the reference and its importance might be included in this first paragraph of this section or as a second paragraph and then refer to the "three essential criteria".

Response: As above in response 3 to Reviewer 3's comments, we have rephrased this paragraph on page 6:

'Our search yielded 14 eligible reference documents. None were obtained through database searches of MEDLINE and EMBASE, and all were obtained from 'grey literature' sources. These statements included one international and 13 national/regional guidelines.

The International Ethical Guidelines for Health-related Research Involving Humans were first released in 1982 and have undergone several revisions, most recently in 2016 (18). These international guidelines are a collaboration between the Council for International Organisations of Medical Sciences (CIOMS) and the World Health Organisation (WHO), and provide a global framework of ethical principles to guide the conduct of all health-related research involving human participants. Guideline 10 pertains to waiver of consent. In addition to the CIOMS international guideline, we identified 1 regional (European Union) and 12 national guidelines applicable in 39 countries (Figure 1). These documents and their intended scope are detailed in Table 1.'

REVIEWER 4

(1) Describe several specific examples of clinical research that received a waiver of informed consent and fully met the three criteria in the WHO document.

Response: We have added to paragraph 3 of the Discussion to make reference to examples of clinical research that utilised waiver of consent: *'Accordingly, clinical research involving waiver of consent is gaining increasing awareness and has been successfully and appropriately utilised in a number of clinical studies (Table 4).'* We have included the following table (Table 4 on page 24) to provide 3 clear and diverse examples.

Table 4. Examples of completed clinical research studies utilising waiver of informed consent.

**Study name**	**Study characteristics**	**Primary indication(s) for waiver of consent**	**Study impact**
Personalised cooler dialysate for patients receiving maintenance haemodialysis (MyTEMP): a pragmatic, cluster-randomised trial (45)	Large pragmatic, cluster-randomised trial conducted in haemodialysis centres in Canada, comparing standard temperature dialysate (36.5ºC) versus personalised cooler dialysate temperature (0.5ºC below the patient's body temperature with a minimum of 35.5ºC and maximum of 36.5ºC) among patients receiving maintenance haemodialysis	- A minimal risk study<br>- The intervention compared two low-risk practice variations<br>- Randomisation occurred at the cluster level (ie. haemodialysis centres were randomised as clusters) and attempts to consent individuals would lead to contamination and severe bias in the study design	4.3 million haemodialysis treatments were provided to 15,413 patients across 84 haemodialysis centres in this large pragmatic trial, which demonstrated no difference in major adverse cardiovascular events or intradialytic hypotension events between the intervention arms
Comparative effectiveness of high-dose versus standard-dose influenza vaccination on numbers of US nursing home residents admitted to hospital: a cluster-randomised trial (46, 47)	Large pragmatic, comparative effectiveness, cluster-randomised trial compared high-dose versus standard-dose influenza vaccination in nursing home residents in the USA to assess the effect on hospitalisations, mortality and functional decline	- A minimal risk study<br>- A population at high risk of the outcome being assessed who are typically poorly represented in traditional randomised controlled trials<br>- Randomisation occurred at the cluster level (ie. nursing homes were randomised as clusters), and attempts to consent individuals would lead to contamination and severe bias in the study design	823 nursing homes with 92,269 residents were included in this large and generalisable trial, which demonstrated that high-dose influenza vaccination reduces the risk of respiratory-related hospital admissions for nursing home residents
Comparative effectiveness of a multifaceted intervention to improve adherence to annual colorectal cancer screening in community health centers: A randomized clinical trial (48, 49)	Patient-level randomised controlled trial of patients in participating community health centres in the USA who were identified from electronic health records and randomised to receive either usual care for colorectal cancer screening (computerised	- A minimal risk study<br>- Informed consent not scientifically feasible without compromising validity of the study in delivering an intervention to improve screening adherence<br>- Assessing a fully representative population (including low socio-	450 patients were identified and randomised to the two arms, with the low-cost multifaceted intervention strategy achieving a much higher rate of adherence to colorectal cancer screening (82.2%) compared with

	reminders to healthcare providers and clinician feedback on screening rates) or a multifaceted intervention to facilitate colorectal cancer screening (mailed reminder letter to patients, mail out of a home faecal occult blood test kit, telephone and text message reminders to patients)	economic groups and ethnic minority groups who are typically poorly represented in randomised controlled trials involving traditional consent models)<br>- All patient information and outcome data collection were obtained from routine care records	the usual care group (37.3%)

(2) The 'declaration' of minimal risk requires further discussion. Risk is a combination of severity of harm and probability of that harm. How do severity and probability work together to define 'minimal' risk to patients in the real world of clinical medicine.

Response: We thank reviewer 4 for the comments on the definition of minimal risk, particularly that it is a combination of both severity and probability of harm. We have added to the discussion of this criterion on page 6:
'Minimal risk' is specifically defined in twelve guidelines, with unifying concepts being that the anticipated probability and magnitude of harm is small. The concept is sometimes expressed as no or minimal additional risk from participation in the research relative to that of not participating (the background risk of the management of the condition in clinical care)'.

We have also added to paragraph 3 of the Discussion on page 10:
'Waiver of consent is of value in situations where the research would not otherwise be feasible for methodological reasons (eg. interventions with cluster-level participation or cluster-level outcomes), where there is no or minimal risk (with 'minimal' referring to both the severity and probability of risk), and where there is anticipated to be significant individual, community and/or societal value and real-world evidence from the research being conducted.'

(2) The authors rightly conclude that waiver of informed consent research may 'facilitate learning health system environments that can provide rigorous assessments of the relative effectiveness of variations in clinical practice.' I am well aware that there are important variations in clinical practice; however, this begs the question of generalizability of findings from studies conducted on limited cohorts with patients that did not receive informed consent.

Response: With regards to generalisability of a study, this is defined by the study's inclusion/exclusion criteria and by the population from which participants are sampled. By nature, studies involving a waiver of consent will always favour a more generalisable population by ensuring inclusion of a broader and larger cohort of patients (including often marginalised and minority group participants) than would otherwise be enrolled if consent were feasible or mandated for the study. This is particularly true of cluster-randomised trials where cluster-based randomisation may necessitate a waiver of consent to avoid contamination and prevent severe bias in study design, and clusters usually contain large populations. Most importantly, by definition, the statements reviewed for this paper emphasise that justification for utilising waiver of consent in clinical research must include that there be 'important social value' for the research being conducted. Included in this concept is that the research is of benefit to society and should generate generalisable research findings.

In order to better articulate the potential of waiver of consent studies in providing more applicable and generalisable evidence, we have added to paragraph 2 of the Introduction on page 4: *'Exploratory randomised trial populations have historically differed substantially from the broader patient population (4-7). These differences stem in part from inclusion and exclusion criteria that are designed both to protect participant safety and to define a population most suited to testing the study hypothesis. However, they also result from the informed consent process itself, which requires participants understand consent material written in technical and legal style (or have a guardian able to do so), and to sufficiently engaged in their healthcare so as to volunteer or accept referral for inclusion in research. These factors result in study populations which differ in important ways from the broader patient population, for example being younger, less comorbid, or with a better prognosis. Contrastingly, comparative effectiveness studies and evaluations of health-service interventions are designed for broader real-world settings and will naturally lead to changes in practice that affect all patients. Such studies will be most informative if they include generalisable populations, are sufficiently large, and minimise bias. Given the barriers that traditional written informed consent models pose to inclusion of a representative patient population, such studies of practices where patient consent is not sought in clinical practice may warrant a similar waiver of research consent to minimise selection and other biases (8-10).'*

As discussed in the response to comment 1 from Reviewer 4, we have now also included 3 examples of studies utilising waiver of consent in Table 4 of the manuscript on page 24. The

first 2 trials described in this table are cluster-randomised trials with greater than 15,000 participants and hence we feel these examples also aide to convey to the reader the potential of waiver of consent to generate hugely generalisable evidence.

(3) Specific examples of how this has been applied in the real world to facilitate learning among health systems would strengthen the manuscript.

Response: Please refer to response 1 to Reviewer 4's comments and our inclusion of specific examples of real-world waiver of consent studies that have been completed.

(4) How has informed-consent-waived research resulted in broad scale learning outside the 'system' where the research was conducted. (5) How has a waiver-of-informed-consent study led to changes in medical guidelines?

Response: Both of these questions relate to examples of waiver of consent research and their relative impact. The examples of waiver of consent studies that we have now included in Table 4 on page 24 describe 3 large and impactful studies and clearly emphasise the potential of waiver of consent in facilitating generalisable studies that have led to broad international practice changes.

VERSION 2 - REVIEW

Reviewer **2**
Name **Jamrozik, Euzebiusz**
Affiliation **University of Oxford, Ethox and the Wellcome Centre for Ethics and Humanities**
Date **06-Feb-2025**
COI

Much improved with appropriate widening of scope compared to the initial version.
